# cirSIRT5 induces ferroptosis in bladder cancer by forming a ternary complex with SYVN1/PHGDH

**DOI:** 10.1038/s41420-024-02163-4

**Published:** 2024-09-02

**Authors:** Weijian Li, Yuxi Ou, Fangdie Ye, Zhang Cheng, Ziang Chen, Quan Zhou, Xiang Yan, Haowen Jiang

**Affiliations:** 1 Department of Urology, Huashan Hospital, Fudan University, Shanghai, China; 2 Fudan Institute of Urology, Huashan Hospital, Fudan University, Shanghai, China; 3 Department of Urology, Pediatric Urolith Center, Children’s Hospital, Zhejiang University School of Medicine, National Clinical Research Center for Child Health, Hangzhou, China; 4 National Clinical Research Center for Aging and Medicine, Fudan University, Shanghai, China; 5 Department of Urology, Jing’an District Central Hospital, Fudan University, Shanghai, China

**Keywords:** Cancer, Biomarkers, Molecular biology

## Abstract

Bladder cancer (BC) represents a prevalent and formidable malignancy necessitating innovative diagnostic and therapeutic strategies. Circular RNAs (circRNAs) have emerged as crucial regulators in cancer biology. In this study, we comprehensively evaluated ferroptosis levels in BC cells utilizing techniques encompassing lipid peroxidation assessment, transmission electron microscopy, and malondialdehyde (MDA) measurement. Additionally, we probed into the mechanistic intricacies by which circRNAs govern BC, employing RNA pull-down, RNA immunoprecipitation (RIP), and immunoprecipitation (IP) assays. Our investigation unveiled circSIRT5, which displayed significant downregulation in BC. Notably, circSIRT5 emerged as a promising prognostic marker, with diminished expression correlating with unfavorable clinical outcomes. Functionally, circSIRT5 was identified as an inhibitor of BC progression both in vitro and in vivo. Mechanistically, circSIRT5 exerted its tumor-suppressive activities through the formation of a ternary complex involving circSIRT5, SYVN1, and PHGDH. This complex enhanced the ubiquitination and subsequent degradation of PHGDH, ultimately promoting ferroptosis in BC cells. This ferroptotic process contributed significantly to the inhibition of tumor growth and metastasis in BC. In addition, FUS was found to accelerate the biogenesis of circSIRT5 in BC. These findings provide valuable insights into the pivotal role of circSIRT5 in BC pathogenesis, underscoring its potential as a diagnostic biomarker and therapeutic target for this malignancy.

## Introduction

Bladder cancer (BC) has emerged as one of the most prevalent malignancies worldwide, accounting for ~3.0% of new cancer cases and 2.1% of cancer-related deaths annually [1, 2]. Urothelial carcinoma represents its most common histological subtype, predominantly comprising non-muscle-invasive BC (NMIBC) and muscle-invasive BC (MIBC). Currently, there remains a dearth of optimal therapeutic strategies for MIBC [3]. Consequently, there is an urgent need for the development of novel diagnostic biomarkers and therapeutic targets to enhance the prognosis of BC patients.

Circular RNA (circRNA) represents a novel class of non-coding RNA, generated via exon back-splicing of precursor mRNA (pre-mRNA) [4]. Distinguished from linear non-coding RNAs, the circular structure confers circRNAs with enhanced stability, rendering them more likely to function persistently within cells [5]. Numerous circRNAs have been identified to exert regulatory roles in various biological processes, particularly in cancer [6]. For instance, circRILPL1 has been implicated in promoting malignant progression in nasopharyngeal carcinoma by activating the Hippo-YAP signaling pathway [7]. Additionally, Yi J et al. reported that CircPVT1 could facilitate ER-positive breast tumorigenesis and drug resistance through its interaction with ESR1 and MAVS [8]. Thus, unraveling the mechanisms underlying bladder cancer malignancy from the perspective of circRNAs holds promise in identifying novel diagnostic markers and therapeutic targets for bladder cancer.

In our present study, we have identified a novel circular RNA (circSIRT5) that exhibits significant downregulation in bladder cancer tissues. Further investigations have revealed its potential as a tumor suppressor, both in vitro and in vivo, and its association with improved prognosis in bladder cancer patients. Mechanistic studies have unveiled that circSIRT5 exerts its anticancer effects by forming a circSIRT5/SYVN1/PHGDH ternary complex, thereby facilitating the ubiquitination and degradation of PHGDH, ultimately promoting ferroptosis in bladder cancer cells and subsequently exhibiting its antitumor activity.

## Results

### Characteristics of circSIRT5 in BC

**Fig. 1:**
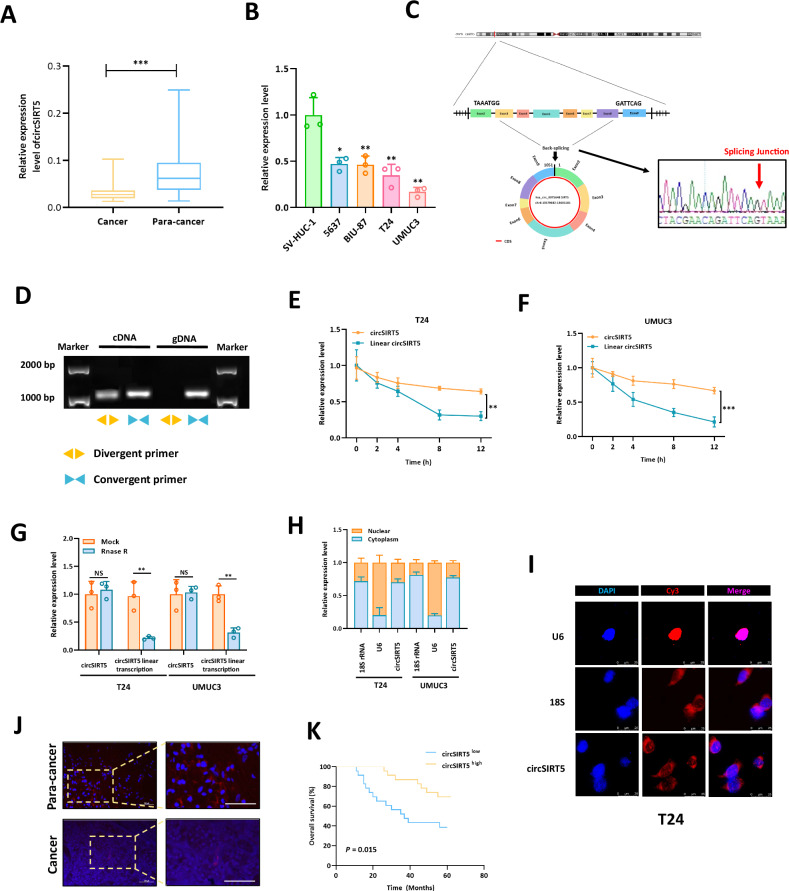
Characteristics of circSIRT5 in bladder cancer (BC). **A** Expression analysis of circSIRT5 in cancer and para-cancer tissues by qRT-PCR. **B** Expression analysis of circSIRT5 in bladder cancer cell lines by qRT-PCR. **C** Schematic representation of the circSIRT5 molecular structure, unveiling its splicing junction sequence through Sanger sequencing. **D** Agarose gel electrophoresis analysis of RT-PCR amplification products using divergent and convergent primers for circSIRT5. **E**, **F** Assessment of the degradation kinetics of circSIRT5 and linear circSIRT5 in T24 and UMUC3 cells upon treatment with Actinomycin D (2 μg/ml) by RT-PCR. **G** Investigation of circSIRT5 and linear circSIRT5’s resistance to RNase R in BC cells, followed by RT-PCR analysis. **H** Examination of circSIRT5 expression levels within the cytoplasm and nucleus through nuclear-cytoplasmic fractionation experiments. (**I**) Subcellular distribution analysis of circSIRT5 using fluorescence in situ hybridization (FISH) experiments. Scale bar: 25 um. **J** FISH analysis revealed the expression level of circSIRT5 in BC tissues. (*N* = 46) **K** Kaplan–Meier survival analysis to assess the survival outcomes of BC patients with high circSIRT5 expression and low circSIRT5 expression groups. *P < 0.05, **P < 0.01, ***P < 0.001.

We identified a group of circRNAs exhibiting significant differential expression in BC through our previous research [9]. Among them, hsa_circ_0075648 (circSIRT5) drew our attention due to its significantly decreased expression in bladder cancer. First, we explored the expression of hsa_circ_0075648 (circSIRT5) in 46 pairs of BC samples using RT-PCR and found a significant downregulation in bladder cancer tissues (Fig. 1A). Additionally, hsa_circ_0075648 (circSIRT5) was found to be significantly lower in bladder cancer cell lines 5637, BIU-87, T24, and UMUC3 compared to normal urothelial cell line SV-HUC-1, consistent with sequencing results (Fig. 1B). Structural analysis revealed that hsa_circ_0075648 is formed by back-splicing of exons 2–9 of the SIRT5 gene, and thus, we named it circSIRT5. Furthermore, sanger sequencing confirmed the sequence of the circSIRT5 back-splicing site, validating its existence (Fig. 1C). To further confirm the circular structure of circSIRT5, we designed specific divergent and convergent primers for RT-PCR and analyzed the RT-PCR products by agarose gel electrophoresis. Results showed that divergent primers amplified cDNA rather than gDNA, indicating the circular structure of circSIRT5 (Fig. 1D). Moreover, circular RNAs are known for their increased stability due to their circular structure. Actinomycin D treatment experiments demonstrated that circSIRT5 had a slower degradation rate relative to linear circSIRT5 in T24 (Fig. 1E) and UMUC3 (Fig. 1F) cells. Additionally, RNase R treatment experiments showed that circSIRT5 exhibited stronger resistance to RNase R compared to its linear circSIRT5 counterpart (Fig. 1G). These results collectively confirmed the increased stability of circSIRT5 relative to linear circSIRT5, indirectly verifying its circular structure.

Furthermore, nuclear-cytoplasmic fractionation experiments (Fig. 1H) and FISH assay (Fig. 1I) revealed that circSIRT5 predominantly localized to the cytoplasm of BC cells. We analyzed the correlation between circSIRT5 and clinical characteristics of BC patients and found associations with tumor grade, tumor size, T stage, N stage, and muscle invasion (Table 1). Additionally, we conducted circSIRT5 expression analysis on tissue samples from our cohort using FISH probes. Our findings revealed a significantly lower expression of circSIRT5 in BC tissues compared to adjacent normal tissues, which was consistent with the results obtained through PCR analysis (Fig. 1J). Kaplan–Meier analysis indicated that BC patients with low circSIRT5 expression had a poorer prognosis compared to those with high circSIRT5 expression (Fig. 1K), suggesting its potential as a prognostic monitoring factor for BC.

**Table. 1:** Correlations between circSIRT5 expression levels and the clinicopathological characteristics of bladder cancer patients.

Characteristics	Number of cases	circSIRT5 expression		*P* value
		Low	High	
Total	46	23	23	
Gender				0.381
Female	6	2	4	
Male	40	21	19	
Grade				0.009*
Low	9	1	8	
High	37	22	15	
Age (years)				0.437
<60	8	3	5	
≥60	38	20	18	
Tumor size (cm)				0.003*
<3	24	7	17	
å =3	22	16	6	
T stage				0.016*
T1-T2	28	10	18	
T3-T4	18	13	5	
N stage				
N0	34	13	21	0.007*
N1	12	10	2	
Muscle invasion				
No	30	11	19	0.013*
Yes	16	12	4	

^*^P < 0.05 is considered significant.

### circSIRT5 inhibits BC cell proliferation, invasion, and migration

**Fig. 2:**
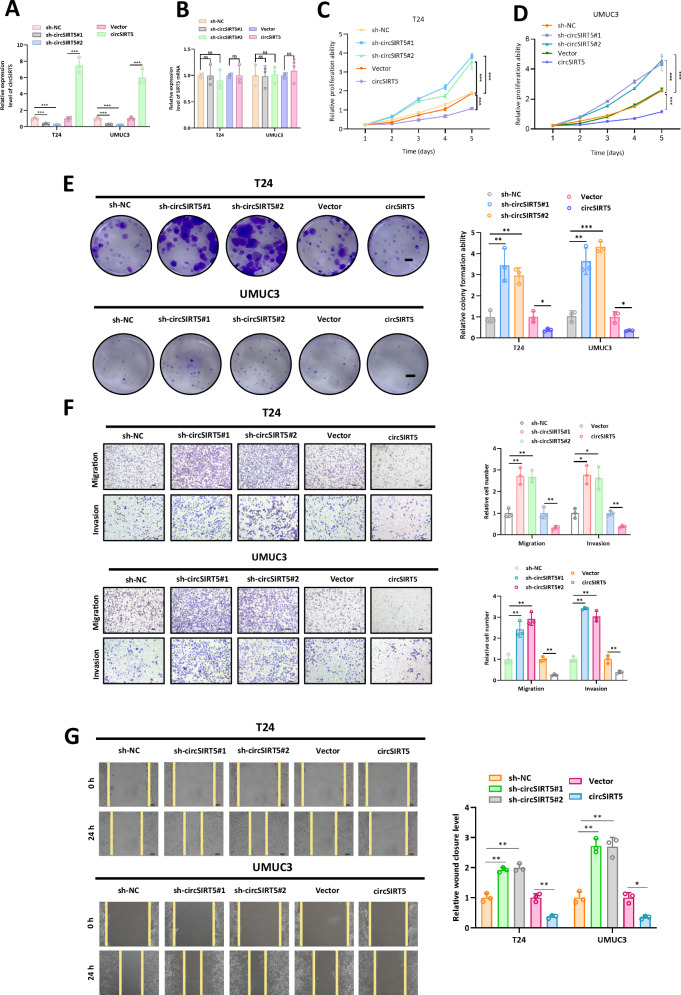
circSIRT5 inhibits BC cell proliferation, invasion, and migration. **A** The efficiency of circSIRT5 overexpression or circSIRT5 silencing was assessed using RT-PCR analysis. **B** The impact of circSIRT5 silencing or overexpression on SIRT5 mRNA expression was analyzed by RT-PCR. The proliferative capacity of T24 (**C**) and UMUC3 (**D**) cells following circSIRT5 silencing or overexpression was assessed using the CCK-8 assay. **E** Changes in the proliferative potential of BC cells following circSIRT5 silencing or overexpression were evaluated via colony formation assay. Scale bar: 20 mm. **F** Alterations in the migratory and invasive capabilities of BC cells following circSIRT5 silencing or overexpression were assessed using the Transwell assay. Scale bar: 100 um. **G** Wound healing assay revealed the migration capacity of BC cells in each group. *P < 0.05, **P < 0.01, ***P < 0.001.

To investigate the impact of circSIRT5 on the malignant phenotype of BC cells, we synthesized specific shRNAs targeting circSIRT5 (sh-circSIRT5#1 and sh-circSIRT5#2), overexpression plasmids for circSIRT5 (circSIRT5), and their respective control groups (sh-NC, Vector). We then established stably transfected BC cell lines through lentiviral transduction. The efficiency of circSIRT5 knockdown and overexpression was validated by RT-PCR, revealing a significant reduction in circSIRT5 expression with sh-circSIRT5#1 and sh-circSIRT5#2, and a marked increase in circSIRT5 expression in the circSIRT5 overexpression cell lines (Fig. 2A). Furthermore, we examined the impact of these interventions on the parental SIRT5 mRNA levels. Results demonstrated that sh-circSIRT5 and circSIRT5 overexpression did not affect SIRT5 mRNA levels (Fig. 2B). CCK-8 assays indicated that the proliferation capacity of BC cells was significantly higher in sh-circSIRT5#1 and sh-circSIRT5#2 groups compared to the sh-NC group, whereas BC cells in the circSIRT5 group exhibited significantly reduced proliferation compared to the Vector group (Fig. 2C, D). Additionally, colony formation assays revealed that circSIRT5 silencing significantly increased BC cell proliferation, while circSIRT5 overexpression significantly reduced BC cell proliferation (Fig. 2E). Moreover, Transwell assays and wound healing assay showed that BC cell migration and invasion abilities were significantly higher in sh-circSIRT5#1 and sh-circSIRT5#2 groups compared to the sh-NC group, whereas circSIRT5 overexpression significantly decreased BC cell migration and invasion abilities (Fig. 2F, G). Collectively, these results indicate that circSIRT5 can inhibit the malignant progression of BC in vitro.

### circSIRT5 directly binds to PHGDH

**Fig. 3:**
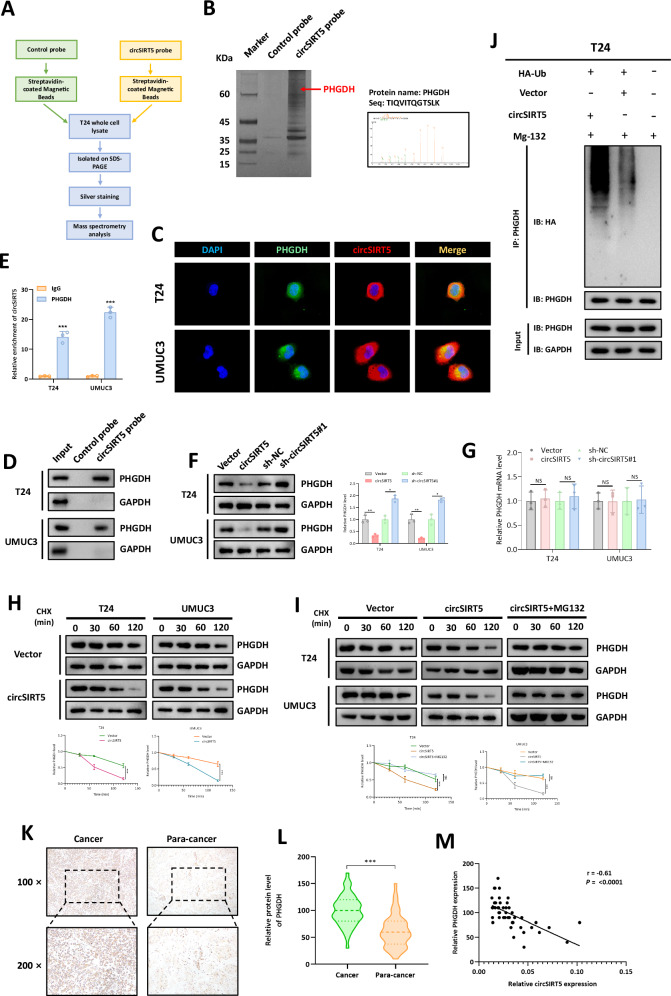
circSIRT5 directly binds to PHGDH and enhances PHGDH degradation via the ubiquitin-proteasome pathway in BC. **A** Schematic depiction of the RNA pull-down assay workflow. **B** Left panel: Silver staining analysis of the RNA pull-down products; Right panel: Representative mass spectrometry data for PHGDH. **C** IF and FISH assays were employed to elucidate the subcellular localization of PHGDH and circSIRT5 in BC cells. **D** Western blot analysis was conducted to assess the levels of PHGDH expression in the RNA pull-down products. **E** RIP assay was carried out to investigate the interaction between PHGDH and circSIRT5. **F** Western blot analysis was performed to determine the levels of PHGDH protein following circSIRT5 silencing or overexpression. **G** RT-PCR analysis was employed to assess the levels of PHGDH mRNA subsequent to circSIRT5 silencing or overexpression. **H** Western blot analysis was utilized to ascertain the protein degradation rate of PHGDH before and after circSIRT5 overexpression, with concurrent cycloheximide (CHX) inhibition of protein synthesis. **I** Western blot analysis was conducted to evaluate the impact of the proteasome inhibitor MG132 (20 μM) on the protein degradation rate of PHGDH. **J** Ubiquitination assay was carried out to investigate the effect of circSIRT5 expression levels on the ubiquitination of PHGDH. **K** IHC analysis on 46 pairs of BC tissues. **L** Quantification analysis of IHC. **M** Correlation between PHGDH IHC scores and circSIRT5 expression levels. *P < 0.05, **P < 0.01, ***P < 0.001.

In order to identify the target through which circSIRT5 exerts its function, we conducted a biotin-labeled RNA pull-down experiment (Fig. 3A). Subsequently, we subjected the pull-down products to silver staining and LC-MS/MS analysis, and one cancer-related protein, PHGDH, particularly piqued our interest (Fig. 3B). FISH-IF experiments revealed the subcellular co-localization of PHGDH and circSIRT5 in BC cells, indicating a spatial basis for their interaction (Fig. 3C). Western Blot analysis of the pull-down products confirmed that PHGDH interacted with the circSIRT5 probe but not the control probe (Fig. 3D). Additionally, RIP (RNA immunoprecipitation) experiments also indicated that circSIRT5 could be immunoprecipitated by PHGDH antibodies (Fig. 3E). These collective findings strongly suggest an interaction between PHGDH and circSIRT5.

### circSIRT5 enhances PHGDH degradation via the ubiquitin-proteasome pathway in BC

The manner in which circSIRT5 regulates PHGDH has captured our attention. Western blot analysis revealed a significant decrease in PHGDH protein levels upon circSIRT5 overexpression, while a notable increase was observed following circSIRT5 silencing (Fig. 3F). Interestingly, RT-PCR analysis indicated that circSIRT5 did not affect PHGDH mRNA levels, suggesting that circSIRT5 regulates PHGDH expression post-transcriptionally (Fig. 3G). Subsequent protein synthesis inhibition experiments using cycloheximide (CHX) demonstrated that circSIRT5 overexpression accelerated PHGDH degradation (Fig. 3H). Furthermore, the addition of the proteasome inhibitor MG132 significantly attenuated circSIRT5-mediated enhancement of PHGDH degradation, suggesting that circSIRT5 promotes PHGDH degradation through the ubiquitin-proteasome pathway (Fig. 3I). In addition, ubiquitination analysis revealed a significant increase in PHGDH ubiquitination levels following circSIRT5 overexpression (Fig. 3J). These results collectively indicate that circSIRT5 reduces PHGDH protein levels by facilitating its ubiquitination-mediated degradation. Furthermore, we conducted IHC analysis on 46 pairs of BC tissues, which showed significantly elevated PHGDH expression levels in BC compared to normal tissues (Fig. 3K, L), consistent with previous studies. Intriguingly, we discovered a significant negative correlation between PHGDH IHC scores in BC tissues and circSIRT5 expression levels (*r* = −0.061, *P* < 0.0001) (Fig. 3M). These findings suggest that circSIRT5 interacts with PHGDH and promotes its ubiquitination-mediated degradation.

### The circSIRT5/ SYVN1/PHGDH complex promotes the degradation of PHGDH

**Fig. 4:**
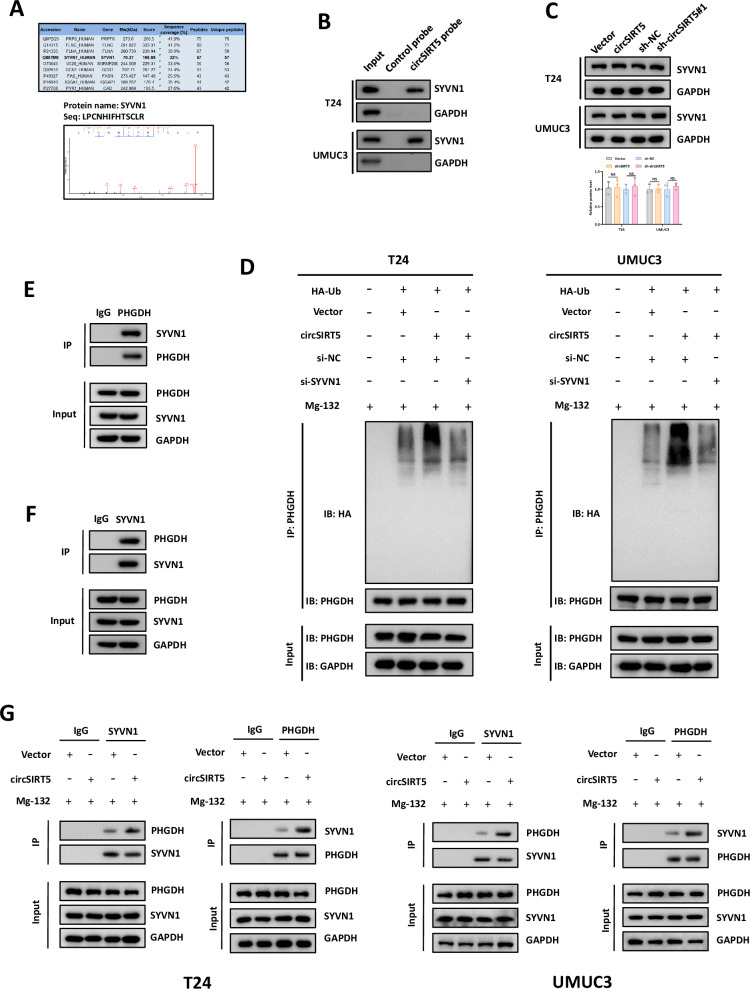
The circSIRT5/ SYVN1/PHGDH complex promotes the degradation of PHGDH. **A** Upper panel: A partial protein list from mass spectrometry analysis of RNA pull-down samples; Lower panel: The mass spectrometry data for SYVN1. **B** Western blot analysis depicting the expression levels of SYVN1 in the RNA pull-down products. **C** Western blot analysis illustrating the impact of circSIRT5 silencing or overexpression on SYVN1 protein levels. **D** Ubiquitination assay investigating the effects of circSIRT5 silencing or overexpression, as well as SYVN1 silencing or overexpression, on the ubiquitination levels of PHGDH. **E** Western blot analysis assessed the levels of SYVN1 protein within the immunoprecipitates obtained using the PHGDH antibody in T24 cells. **F** Western blot analysis was conducted to evaluate the levels of PHGDH protein within the immunoprecipitates obtained using the SYVN1 antibody in the T24 cell. **G** Co-IP experiments revealing the influence of circSIRT5 expression levels on the interaction between SYVN1 and PHGDH in BC cells. *P < 0.05, **P < 0.01, ***P < 0.001.

We delved further into the mechanisms underlying circSIRT5-mediated PHGDH ubiquitination and degradation. Our analysis of proteins from the circSIRT5 pull-down proteins led us to identify the E3 ligase SYVN1 as a significant candidate (Fig. 4A). In the ubiquitination process, the E2 ubiquitin-conjugating enzymes, together with E3 ubiquitin ligases, facilitate the attachment of ubiquitin moieties onto substrate proteins, thereby guiding the substrate proteins towards ubiquitination and subsequent degradation. Consequently, E3 ubiquitin ligases play a pivotal role in selecting specific substrates for ubiquitination reactions [10]. On this basis, we postulated that SYVN1 might participate in the circSIRT5-mediated ubiquitination of PHGDH. Western blot analysis revealed the specific binding of the circSIRT5 probe, as opposed to the control probe, to SYVN1 protein, suggesting an interaction between the two (Fig. 4B). To ascertain the regulatory role of circSIRT5 on SYVN1, we conducted Western blot analysis of SYVN1 expression following circSIRT5 silencing or overexpression. Surprisingly, changes in circSIRT5 expression did not correlate with alterations in SYVN1 expression levels (Fig. 4C). Furthermore, ubiquitination analysis indicated that SYVN1 silencing significantly restored the circSIRT5-mediated promotion of PHGDH ubiquitination (Fig. 4D). Consequently, we hypothesized that circSIRT5, by forming a circSIRT5/SYVN1/PHGDH ternary complex, enhances the interaction between E3 ubiquitin ligase SYVN1 and its substrate PHGDH, thereby facilitating PHGDH ubiquitination and subsequent degradation. To validate our hypothesis, co-IP experiments demonstrated the interaction between SYVN1 and PHGDH in BC cells (Fig. 4E, F). Furthermore, co-IP results revealed a significant enhancement in the interaction between SYVN1 and PHGDH following circSIRT5 overexpression (Fig. 4G). Collectively, these results support the notion that the formation of the circSIRT5/SYVN1/PHGDH ternary complex promotes the ubiquitination and degradation of PHGDH.

### PHGDH is a functional downstream mediator for circSIRT5

**Fig. 5:**
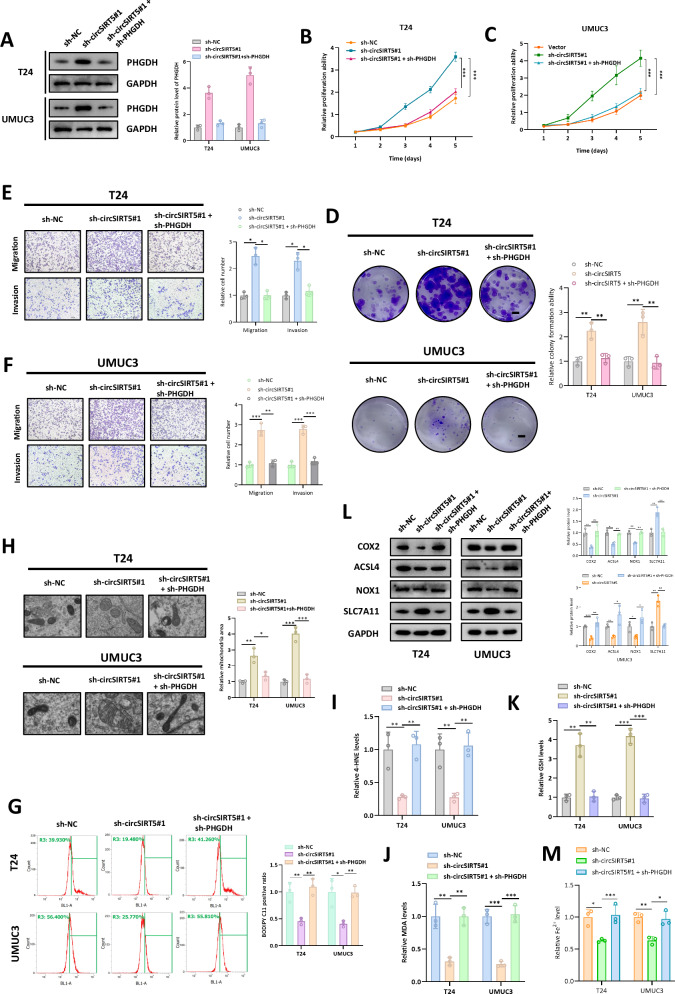
circSIRT5 promotes ferroptosis in BC cells by regulating PHGDH. **A** Protein levels of PHGDH in each experimental group were analyzed using Western blot assays. The proliferative capacity of T24 (**B**) and UMUC3 (**C**) cells in each group was assessed via CCK-8 assays. **D** The proliferative potential of BC cells in each group was determined by colony formation assay. Scale bar: 20 mm. Transwell assays were employed to evaluate changes in the migratory and invasive abilities of T24 (**E**) and UMUC3 (**F**) cells. Scale bar: 100 um. **G** Lipid peroxidation levels in BC cells of each treatment group were analyzed using the C11-BODIPY probe. Erastin: 5 μm. **H** Transmission electron microscopy was used to assess mitochondrial area in BC cells of the respective groups. Levels of lipid peroxidation markers, 4-HNE (**I**), MDA (**J**), and GSH (**K**), were measured in each treatment group. **L** Expression levels of ferroptosis-related proteins in BC cells of each treatment group were determined by Western Blot analysis. **M** Fe 2+ levels in BC cells. *P < 0.05, **P < 0.01, ***P < 0.001.

To elucidate the role of PHGDH in the regulation of BC by circSIRT5, we synthesized sh-PHGDH to silence PHGDH and conducted rescue experiments. Western blot analysis revealed that PHGDH silencing significantly reduced the elevated levels of PHGDH expression induced by circSIRT5 silencing (Fig. 5A). CCK-8 analysis demonstrated that PHGDH silencing in BC cells significantly restored the proliferative capacity enhancement caused by circSIRT5 silencing (Fig. 5B, C). Moreover, colony formation assays showed a significant decrease in the proliferative capacity of BC cells in the “sh-circSIRT5 + sh-PHGDH” group compared to the “sh-circSIRT5” group (Fig. 5D). Additionally, Transwell analysis indicated that PHGDH silencing partially rescued the increased migratory and invasive abilities of BC cells induced by circSIRT5 silencing (Fig. 5E, F). These findings collectively suggest that circSIRT5 inhibits BC progression through PHGDH.

### circSIRT5 promotes ferroptosis in BC cells by regulating PHGDH

Prior research has demonstrated that PHGDH can regulate ferroptosis in BC [11]. Therefore, we investigated the impact of circSIRT5 on the level of ferroptosis in BC cells. Lipid peroxidation is a critical feature of ferroptosis, and we initially assessed lipid peroxidation levels in BC cells. Using the C11-BODIPY probe, we employed flow cytometry to discover that circSIRT5 silencing significantly reduced cellular lipid peroxidation levels, which could be rescued by PHGDH silencing (Fig. 5G). Prior studies have indicated that cells undergoing ferroptosis exhibit mitochondrial shrinkage and reduced volume [12]. We analyzed mitochondrial volume in BC cells from each treatment group using transmission electron microscopy. The results demonstrated that BC cells in the circSIRT5 silencing group exhibited significantly higher mitochondrial volumes compared to the control group, and PHGDH silencing partially reversed the increased mitochondrial volume induced by circSIRT5 silencing (Fig. 5H). Furthermore, the lipid peroxidation markers 4-HNE and MDA significantly decreased after circSIRT5 silencing, and PHGDH silencing partially restored the decreased levels of 4-HNE and MDA (Fig. 5I, J). GSH, as one of the most common intracellular reducing agents, significantly increased in sh-circSIRT5 BC cells, and GSH levels were partially restored in the “sh-circSIRT5+sh-PHGDH” group (Fig. 5K). Additionally, Western blot analysis revealed significant decreases in ferroptosis-related markers COX2, ACSL4, and NOX1 following circSIRT5 silencing, which were partially reversed by PHGDH silencing. Conversely, the ferroptosis-associated factor SLC7A11 significantly increased after circSIRT5 silencing, and this increase could be rescued by PHGDH silencing. These results collectively suggest that circSIRT5 can promote ferroptosis in BC by modulating PHGDH, thereby exerting its anticancer effects in BC.

### circSIRT5 inhibits the growth and metastasis of BC in vivo

**Fig. 6:**
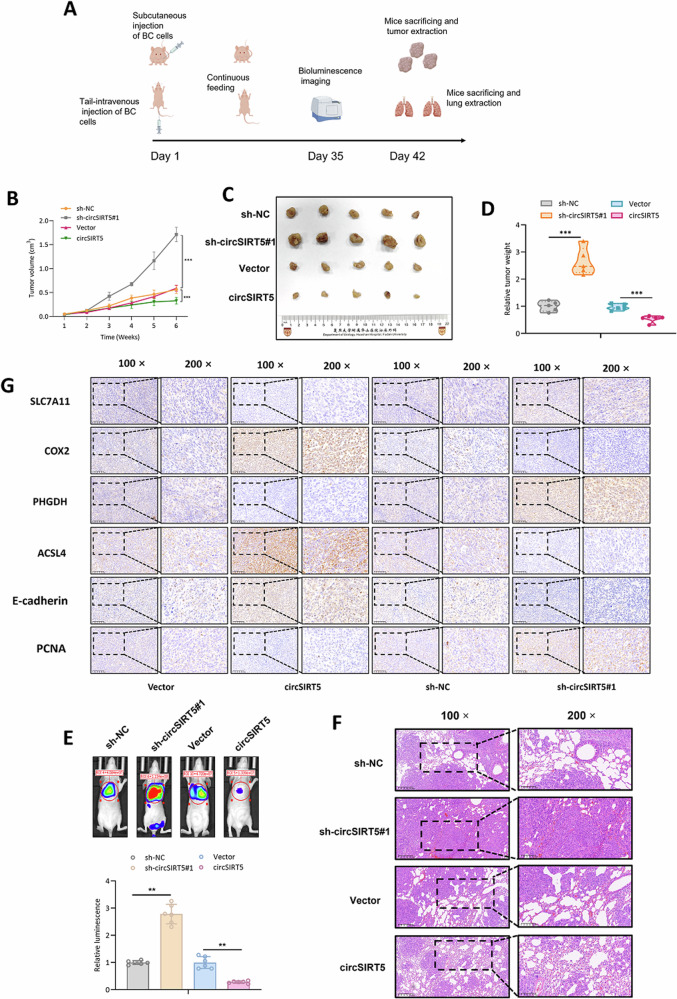
circSIRT5 inhibits the growth and metastasis of BC in vivo. **A** Schematic representation of the experimental workflow for animal studies (Drawn by Figdraw). **B** Tumor volumes of mice in each group were measured and calculated per 7 days. **C** Photographs of excised tumors from mice in the respective groups following euthanasia. **D** Weights of excised tumors from mice in each group following euthanasia. **E** In vivo imaging analysis depicting the extent of lung metastasis in mice from different groups. **F** H&E staining to assess the degree of tumor infiltration in lung tissues of mice from each group. **G** IHC analysis of the expression levels of SLC7A11, COX2, PHGDH, and ACSL4 in tumor tissues of mice from each group. *P < 0.05, **P < 0.01, ***P < 0.001.

In order to investigate the role of circSIRT5 in vivo in bladder cancer (BC), we established subcutaneous tumor xenograft and tail vein lung metastasis models in nude mice (Fig. 6A). In the subcutaneous tumor xenograft model, we observed that the tumor volume was significantly higher in the circSIRT5 silencing group compared to sh-NC, while the tumor volume was markedly reduced in the circSIRT5 overexpression group compared to the Vector group (Fig. 6B, C). Additionally, we found that the tumor weight in the sh-circSIRT5 group was higher than in the sh-NC group, while the tumor weight in the circSIRT5 overexpression group was lower than in the Vector group (Fig. 6D). In the tail vein lung metastasis model, the level of lung metastasis was significantly elevated in the sh-circSIRT5 group compared to the sh-NC group, whereas in the circSIRT5 overexpression group, the level of lung metastasis was significantly lower than in the Vector group (Fig. 6E). Further confirmation was provided by H&E staining of lung tissues (Fig. 6F). Furthermore, immunohistochemical staining analysis revealed that in the tumor tissues of the sh-circSIRT5 group, the expression levels of PHGDH and SLC7A11 were significantly higher than in the sh-NC group, whereas in the circSIRT5 overexpression group, the expression levels of PHGDH and SLC7A11 were markedly lower than in the Vector group. COX2 and ACSL4 expression in the tumor tissues of the sh-circSIRT5 group was significantly lower than in the sh-NC group, and in the circSIRT5 overexpression group, they were significantly higher than in the Vector group (Fig. 6G). These results collectively suggest that circSIRT5 can inhibit the growth and metastasis of BC in vivo.

### FUS regulates the biogenesis of circSIRT5

**Fig. 7:**
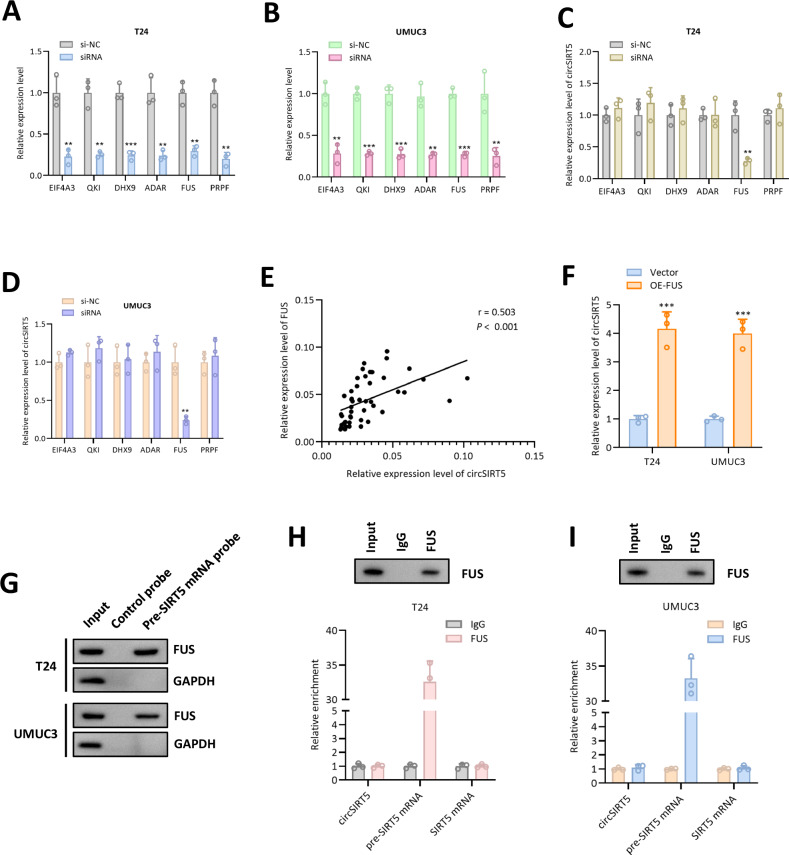
FUS regulates the biogenesis of circSIRT5. RT-PCR assay revealed the expression level of each gene in T24 (**A**) and UMUC3 (**B**). RT-PCR assay revealed the expression level of circSIRT5 in T24 (**C**) and UMUC3 (**D**). **E** RT-PCR assay revealed the relationship between the expression level of circSIRT5 and FUS. **F** RT-PCR assay revealed the expression level of circSIRT5 after FUS overexpression. **G** Western blot assay revealed that FUS could be pulled down by pre-SIRT5 mRNA probes. RIP assay demonstrated significant enrichment of pre-SIRT5 mRNA in FUS antibody immunoprecipitates in T24 (**H**) and UMUC3 (**I**) cells.

The regulatory factors involved in circSIRT5 generation piqued our interest. Previous literature has reported that splicing factors can partake in the back-splicing of circRNA, thereby impacting circRNA generation [13]. To investigate the regulatory factors involved in circSIRT5 back-splicing, we designed specific siRNAs targeting six common RNA splicing factors (EIF4A3, QKI, DHX9, ADAR, FUS, and PRPF). RT-PCR experiments confirmed the efficiency of gene silencing by siRNAs (Fig. 7A, B). Furthermore, we observed that among these six RNA splicing factors, only FUS silencing led to a reduction in circSIRT5 expression levels (Fig. 7C, D). To ascertain the correlation between FUS and circSIRT5 expression levels, we performed PCR and found a significant positive correlation in bladder cancer tissues (Fig. 7E). Additionally, we designed an exogenous FUS overexpression plasmid, and PCR results demonstrated a significant increase in circSIRT5 expression levels following FUS overexpression (Fig. 7F). Research has indicated that FUS can bind to pre-mRNA to regulate circRNA back-splicing, thereby modulating circRNA biogenesis [14]. Consequently, we designed specific probes for pre-SIRT5 mRNA in pull-down experiments, revealing that FUS could be pulled down by pre-SIRT5 mRNA probes, but not by control probes (Fig. 7G). Furthermore, RIP experiments demonstrated significant enrichment of pre-SIRT5 mRNA, rather than circSIRT5 or SIRT5 mRNA, in FUS antibody immunoprecipitates (Fig. 7H, I). These results collectively indicate that FUS can associate with pre-SIRT5 mRNA and promote the generation of circSIRT5.

## Discussion

Since the discovery of circRNA, an increasing number of circRNAs have been identified as key regulators in cancer [15]. Among these, the emerging area of circRNAs directly interacting with proteins to modulate cancer progression has gained prominence [16]. In our study, we initially observed a significant downregulation of circSIRT5 expression in BC and its correlation with the prognosis of BC patients, suggesting its potential as a prognostic biomarker. Further investigations revealed that circSIRT5 exerts its anticancer effects in BC by modulating PHGDH to promote ferroptosis.

D-3-phosphoglycerate dehydrogenase (PHGDH) is a crucial rate-limiting enzyme in the de novo serine synthesis pathway, catalyzing the conversion of 3-phosphoglycerate (3-PG) produced from glycolysis into 3-phosphohydroxypyruvate (3-PHP). PHGDH is frequently overexpressed in tumors through mechanisms such as gene amplification, transcriptional regulation, degradation, and alterations in stability, thereby promoting cancer progression [17]. Recent studies have demonstrated the involvement of PHGDH in the development of various cancers, including esophageal cancer [18], colorectal cancer [19], and hepatocellular carcinoma [20]. Notably, recent research has shown that PHGDH can regulate ferroptosis, thereby influencing enzalutamide resistance in prostate cancer [21] and malignant progression in bladder cancer [11]. Therefore, we further explored whether circSIRT5 regulates ferroptosis mediated by PHGDH in bladder cancer to exert its anticancer effects. Excitingly, silencing circSIRT5 significantly inhibited ferroptosis in BC cells, and silencing PHGDH partially rescued this effect, suggesting that circSIRT5 can modulate the ferroptosis process in BC by regulating PHGDH.

Ubiquitin (Ub) is a protein involved in marking target proteins for degradation within the cell. Ub is initially activated by E1 ubiquitin-activating enzymes and then transferred to E2 ubiquitin-conjugating enzymes. Subsequently, Ub-loaded E2 conjugating enzymes, through interaction with E3 ubiquitin ligases and substrate proteins, guide the substrate protein to the proteasome for degradation, a process known as ubiquitination [10]. Ubiquitination is widely implicated in various cellular processes, especially in cancer [22]. In recent years, as circRNA research has advanced, researchers have found that circRNAs can regulate the ubiquitination of target proteins, affecting their stability [23]. In this study, we discovered that circSIRT5 can modulate the ubiquitination level of PHGDH. Further investigation identified SYVN1 as the E3 ubiquitin ligase responsible for circSIRT5-mediated ubiquitination and degradation of PHGDH. We found that circSIRT5 promotes the formation of the circSIRT5/SYVN1/PHGDH ternary complex, facilitating the ubiquitination and degradation of PHGDH.

## Conclusion

**Fig. 8:**
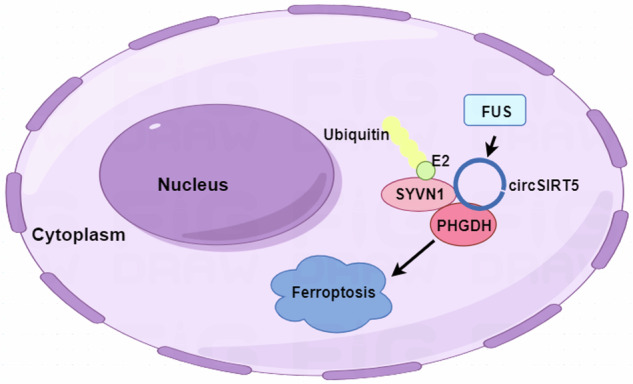
The circSIRT5/SYVN1/PHGDH ternary complex facilitates the ubiquitination and degradation of PHGDH, thereby promoting the schematic representation of ferroptosis in bladder cancer cells (Drawn by Figdraw).

In this study, we observed significant downregulation of circSIRT5 in BC, with its expression significantly correlated with patient prognosis. Further investigations revealed that circSIRT5 promotes the ubiquitination and degradation of PHGDH by forming the circSIRT5/SYVN1/PHGDH ternary complex, thereby facilitating ferroptosis in BC cells and acting as a tumor suppressor (Fig. 8). The elucidation of circSIRT5’s functions holds promise for providing potential prognostic biomarkers and therapeutic targets in BC.

## Materials and methods

### Patient and tissue specimen collection

The collection of human tissue samples adhered to the principles outlined in the Declaration of Helsinki and received approval from the Board and Ethics Committee of Huashan Hospital, Fudan University (Approval No. KY2011-009). Patients with a prior history of other cancers or who had undergone chemotherapy or radiation therapy prior to surgery were excluded from the study. In total, 46 BC patients who underwent radical cystectomy were included in our cohort, with each patient contributing a pair of tissue samples (one bladder cancer tissue and one adjacent normal urothelial tissue). Prior to tissue sample collection, written informed consent was obtained from each patient. All collected samples were divided into two portions: one was promptly frozen in liquid nitrogen and stored at −80 °C, while the other was fixed in formalin and stored at room temperature.

### Cell culture

The UMUC3, T24, 5637, BIU-87, and SV-HUC-1 urothelial cell lines were procured from the Chinese Academy of Sciences (Shanghai, China). UMUC3 cells were cultured in high-glucose Dulbecco’s modified Eagle’s medium (DMEM) (Gibco, USA), while T24, 5637, and BIU-87 cells were cultured in RPMI-1640 medium, SV-HUC-1 was cultured in F12K medium. Each culture medium was supplemented with 10% fetal bovine serum (FBS), 100 units/mL of penicillin, and 100 mg/mL of streptomycin. Cells were maintained in a humidified atmosphere containing 5% CO_2_ at 37 °C.

### Cell counting kit-8 (CCK-8) and colony formation assay

For each experimental group, cells subjected to various treatments were seeded into 96-well plates at a density of 1000 cells per well in 200 μL of medium containing 10% FBS. At specific time points, the medium in each well was replaced with 100 μL of fresh medium containing 10 μL of CCK-8 reagent (Beyotime, China). After incubation for a defined period, the absorbance at 450 nm of each well was measured using a microplate reader (BioTek, USA). For the colony formation assay, cells from different treatment groups were seeded into 12-well plates at a density of 100 cells per well and allowed to grow in a culture medium containing 10% FBS for ~7–10 days. Following removal of the culture medium, cells were washed twice with PBS and fixed with 4% paraformaldehyde at room temperature for 1 h. Subsequently, cells were stained with 0.1% crystal violet for 15 min at room temperature. Finally, cell colony numbers in each well were captured and analyzed under a microscope.

### RNA extraction, reverse transcription, and quantitative real-time PCR analysis (qRT-PCR)

The procedures for RNA extraction, reverse transcription, and qRT-PCR were conducted following previously established methods [24]. Total RNA from cells and tissues was extracted using Trizol reagent (Invitrogen, USA). Subsequently, the extracted RNA was reverse transcribed into cDNA using the Prime Script RT kit (Toyobo, Japan), following the manufacturer’s instructions. qRT-PCR analysis was then performed using the SYBR Green Mix kit (Takara, Japan), according to the manufacturer’s guidelines. GAPDH was employed as an internal reference. Primer sequences for all genes are provided in Table S1.

### Nuclear-cytoplasmic fractionation

Nuclear-cytoplasmic fractionation was performed following previously reported methods [25]. Cell nuclei and cytoplasmic components were separated using the PARIS™ Kit (Thermo Fisher Scientific, USA) according to the manufacturer’s instructions. Subsequently, RNA expression levels in each fraction were analyzed by RT-PCR.

### Lentivirus preparation and infection

The lentiviruses employed in this study were procured from RiboBio (Guangzhou, China). These included shRNAs designed for the specific silencing of circSIRT5, namely, sh-circSIRT5#1 and sh-circSIRT5#2, as well as their corresponding negative control, sh-NC. Overexpression plasmids for circSIRT5 and empty vector controls were also included. UMUC3 and T24 cells were subjected to lentiviral infection and subsequently subjected to puromycin selection at a concentration of 10 µM for 1 week to establish stably transfected cell lines.

### Lipid peroxidation assay

The assessment of lipid peroxidation levels in cells was conducted using the C11-BODIPY probe, following the manufacturer’s recommendations (Thermo Fisher Scientific, USA). Specifically, cells were treated with a 5 μM C11-BODIPY probe for 30 min, followed by two washes with PBS and resuspension. Subsequently, the levels of intracellular lipid peroxidation were analyzed using a flow cytometer (FACSCanto™ II, BD Biosciences).

### Malondialdehyde (MDA) assay

The assessment of MDA levels was conducted following previously reported methods [26]. According to the manufacturer’s instructions, we utilized the lipid peroxidation detection kit (MDA) (Abcam, USA) to assess the relative levels of MDA in cell lysates from different treatments. In brief, mixtures were prepared by combining BC cells subjected to various treatments with specific reagents from the kit. Subsequently, after a series of incubation, cooling, and centrifugation steps, the samples were subjected to absorbance measurements using a microplate reader. The relative levels of MDA were calculated based on a standard curve.

### Glutathione (GSH), 4-hydroxynonenal (4-HNE) assay

In the measurement of Glutathione (GSH) levels, the assay was performed following previously reported methods [27]. The relative concentration of GSH was determined using the Glutathione Assay Kit (Beyotime, China) as per the manufacturer’s instructions, employing a kinetic assay to measure GSH levels indirectly by spectrophotometrically monitoring the absorbance at 412 nm as a result of the reaction with 5-thio-2-nitrobenzoic acid.

For the assessment of 4-hydroxynonenal (4-HNE) levels, reference was made to prior research [28]. The levels of 4-HNE were detected using the Lipid Peroxidation (4-HNE) Assay Kit (Abcam, USA), and the measurements were carried out according to the manufacturer’s guidelines.

### Immunohistochemistry (IHC)

IHC was performed according to previously established protocols [29]. In brief, tissue specimens were fixed in 4% paraformaldehyde, then incubated with primary antibodies against SLC7A11, COX2, PHGDH, and ACSL4, followed by incubation with biotinylated secondary antibodies. Microscopic images were captured and analyzed subsequently. IHC scoring was determined based on the percentage of positively stained cells (0–100%) and the immunostaining intensity (0 = negative; 1 = weak; 2 = moderate; 3 = strong). The antibodies utilized in this study are listed in Table S1.

### Immunofluorescence (IF) and fluorescence in situ hybridization (FISH)

T24 and UMUC3 cells were fixed in 4% paraformaldehyde. Following cell fixation, anti-PHGDH antibodies were applied to block the cells, and subsequent washes were performed to remove unbound antibodies. Cell nuclei were highlighted by staining with 4’,6-diamidino-2-phenylindole (DAPI, Invitrogen). Cellular observation and image capture were carried out using a confocal fluorescence microscope (Olympus, Japan).

For the FISH experiments, a FISH kit from RiboBio (Guangzhou, China) was employed following the manufacturer’s instructions. In essence, Cy3-labeled circSIRT5 probes were synthesized by RiboBio (Guangzhou, China) and were used to target specific RNA molecules. Cellular nuclei were counterstained with DAPI. Cellular visualization and image capture were performed using laser confocal microscopy.

### Immunoprecipitation (IP)

The cells from each group were lysed using IP lysis buffer (Beyotime, China), followed by centrifugation for 30 min at 4 °C and 12,000 rpm to separate the whole-cell lysates. Pre-clearing of the lysates to eliminate nonspecific binding between proteins and magnetic beads was achieved using Protein A/G magnetic beads (Thermo Scientific Fisher, USA). The whole-cell lysates were then divided into Input, IgG, and IP groups. Specified antibodies and the cell lysates were separately incubated overnight at 4 °C. Subsequently, Protein A/G magnetic beads were incubated with the antibody-protein complexes overnight at 4 °C. Magnetic separation of the beads from the lysates was performed using a magnetic rack, and the antibody-protein complexes were eluted from the beads using elution buffer for subsequent analysis by Western Blot. Antibody details are provided in Table S1.

### RNA immunoprecipitation (RIP) assay

The RIP assay was conducted following the manufacturer’s instructions, employing the Magna RIP RNA-Binding Protein Immunoprecipitation Kit (Millipore, USA). qRT-PCR was utilized to assess RNA expression levels. IgG was used as a negative control.

### Transmission electron microscopy (TEM)

The cells from each group were collected and washed repeatedly with PBS (1 × 10^7^ cells per group). After centrifugation, the cells were mixed with agarose and fixed with 1% OsO_4_ (Ted Pella Inc, USA). Subsequently, multiple ethanol dehydration steps were performed on the samples at room temperature using varying concentrations of ethanol. The resin-infiltrated and embedded samples were polymerized in an oven at 65 °C and then sectioned into thin slices using an ultramicrotome (Leica, Germany). Finally, cellular mitochondria were observed, and images were captured using a transmission electron microscope (HT7800/HT7700, HITACHI, Japan).

### Animal study

Four-week-old female BALB/c nude mice used in this study were obtained from SLARC (Shanghai, China). In the subcutaneous tumor model, the nude mice were randomly divided into groups of six each. T24 cell lines stably expressing different constructs (sh-NC, sh-circSIRT5, Vector, circSIRT5) were subcutaneously injected into the backs of the mice at a concentration of 5 × 10^6^ cells per mouse. Tumor dimensions (length, L, and width, W) were measured with calipers every 7 days, and tumor volume (V) was estimated using the formula V = (W^2^ × L)/2. After 42 days post-cell injection, the mice were euthanized, tumors were excised, and their weights were recorded.

In the lung metastasis model, nude mice were randomly divided into groups of six each. Different group of T24 cell lines expressing luciferase were intravenously injected into the mice at a concentration of 1 × 10^6^ cells. After 35 days, luciferin was intraperitoneally injected into the mice, and tumor lung metastasis was observed using an In Vivo Imaging System. After 42 days post-injection, the mice were euthanized, and lung tissues were subjected to Hematoxylin and eosin (H&E) staining for analysis (Fig. 6A).

### Western blot analysis

Total cellular proteins were extracted using RIPA lysis buffer (Beyotime, China) following the manufacturer’s recommendations. After determining the protein concentrations using the BCA assay kit (Thermo Scientific, USA), equal amounts of protein from each group were separated by SDS-PAGE gel electrophoresis and then transferred onto a 0.45 μm polyvinylidene fluoride (PVDF) membrane. Subsequently, the PVDF membrane was blocked with 5% skim milk (1 h, room temperature), followed by overnight incubation at 4 °C with the designated primary antibodies. After washing (three times, 10 min each), the PVDF membrane was incubated with secondary antibodies at room temperature for 1 h. Following another round of washing (three times, 10 min each), chemiluminescence was detected using a chemiluminescence assay kit (Millipore, USA), and images were acquired using the BioSpectrum 600 imaging system. Details of the antibodies used in this study are listed in Table S1.

### RNA pull-down assay

The RNA pull-down assay was conducted following previously established methods [30]. The PureBinding™ RNA-Protein pull-down kit (GENESEED, China) was employed according to the manufacturer’s instructions. In brief, a biotinylated circSIRT5 probe or a negative control probe (GenePharma, China) was incubated with streptavidin-coated magnetic beads for 4 h. Subsequently, cell lysates were incubated with the probe-bead complexes for 12 h at 4 °C. Magnetic separation was used to isolate the beads from the supernatant. Protein immunoblotting and mass spectrometry analysis were employed to identify proteins specifically bound to the circSIRT5 probe.

### Liquid chromatography–tandem mass spectrometry (LC-MS/MS)

The analyzed samples were treated with reaction buffer (1% SDC/100 mM Tris-HCl, pH = 8.5/10 mM TCEP/40 mM CAA) and incubated at 95 °C for 10 min. Protein denaturation, reduction, and alkylation were carried out in a one-step process. The supernatant was collected after centrifugation, and an equal volume of ultrapure water was added for dilution. Trypsin was added at a mass ratio of 1:50 (enzyme to protein), and the mixture was incubated with shaking overnight at 37 °C for enzymatic digestion. On the following day, TFA was added to terminate the digestion, and the supernatant was collected by centrifugation (12,000×*g*). Desalting was performed using a homemade SDB desalting column, followed by vacuum drying and storage at −20 °C. Mass spectrometry data were acquired using a Q Exactive HF mass spectrometer coupled to an UltiMate 3000 RSLCnano liquid chromatography system.

### Statistical analysis

Student’s *t*-tests were employed to ascertain the statistical significance of intergroup differences. Chi-square tests were utilized to analyze the correlation between circSIRT5 expression levels within tumor tissues of BC patients and various clinical-pathological parameters. Survival analysis was conducted using the Kaplan–Meier assay, and the significance of differences in survival rates among groups was determined by the log-rank test. Data analysis was primarily carried out using SPSS 19.0 software (Chicago, USA) and GraphPad Prism 8 software (GraphPad, USA). Each experiment was independently repeated at least three times. A significance level of *P* < 0.05 was considered statistically significant

### Supplementary information

Table S1

Original data file

## Data Availability

The datasets used and/or analyzed during the present study are available from the corresponding author on reasonable request.
