# Supplementary material for: cirSIRT5 induces ferroptosis in bladder cancer by forming a ternary complex with SYVN1/PHGDH
Source: Cell Death Discov. 2024 Sep 2;10:391. doi: 10.1038/s41420-024-02163-4 (PMC11369169; doi:10.1038/s41420-024-02163-4)
Supplement: Supplementary file 1 — Table S1 [file 41420_2024_2163_MOESM1_ESM.docx]

| Table S1 Supplementary information of primers, antibodies and probes used in this study | |
| --- | --- |
| Name | Information |
| circSIRT5 (Divergent) | F：ACCCCAGCTACGAACAGATT |
|  | R：ATCTGGAGAGGTCGCATCAG |
| circSIRT5 (Convergent) | F：TAAATGGAAATGTTTTCTAAC |
|  | R：GCTGAACAGTGGCTCCAAATTA |
| GAPDH mRNA primer | F：TGTTGCCATCAATGACCCCTT |
|  | R：CTCCACGACGTACTCAGCG |
| PHGDH mRNA primer | F：CACGACAGGCTTGCTGAATGA |
|  | R：CTTCCGTAAACACGTCCAGTG |
| SIRT5 mRNA primer | F：GCCATAGCCGAGTGTGAGAC |
|  | R：CAACTCCACAAGAGGTACATCG |
| circSIRT5 FISH probe | CCAGCTACGAACAGATTCAGTAAATGGAA |
| U6 FISH probe | TTTGCGTGTCATCCTTGCG |
| 18S FISH probe | CT+TCCT+TGGATGTGGT+AGCCGT+TTC |
| sh-circSIRT5#1 shRNA | CGAACAGATTCAGTAAATGGA |
| sh-circSIRT5#2 shRNA | ATTCAGTAAATGGAAATGTTT |
| si-SYVN1 siRNA | GCCGCAUUGUCUCUCUUAUTT |
|  | AUAAGAGAGACAAUGCGGCTT |
| sh-PHGDH shRNA | CCGGAGGTGATAACACAGGGAACATCTCGAGA TGTTCCCTGTGTTATCACCTTTTTT |
| SLC7A11 antibody | IHC 1:500;WB 1:2000; Protentech: Cat No : 26864-1-AP |
| COX2 antibody | IHC 1:400;WB 1:1000; Protentech: Cat No : 27308-1-AP |
| PHGDH antibody | IHC 1:2000;IP;WB 1:1000;IF 1:500; Protentech: Cat No : 14719-1-AP |
| ACSL4 antibody | IHC 1:200;WB 1:2000; Protentech: Cat No : 22401-1-AP |
| SYVN1 antibody | IP 1:500;WB 1:2000; Protentech: Cat No : 13473-1-AP |
| HA antibody | WB 1:5000; Protentech: Cat No : 51064-2-AP |
| GAPDH antibody | WB 1:5000; Protentech: Cat No : 10494-1-AP |
| NOX1 antibody | WB 1:1000; Protentech: Cat No : 17772-1-AP |
