# Supplementary figures and images for: cirSIRT5 induces ferroptosis in bladder cancer by forming a ternary complex with SYVN1/PHGDH
Source: Cell Death Discov. 2024 Sep 2;10:391. doi: 10.1038/s41420-024-02163-4 (PMC11369169; doi:10.1038/s41420-024-02163-4)

Fig 3

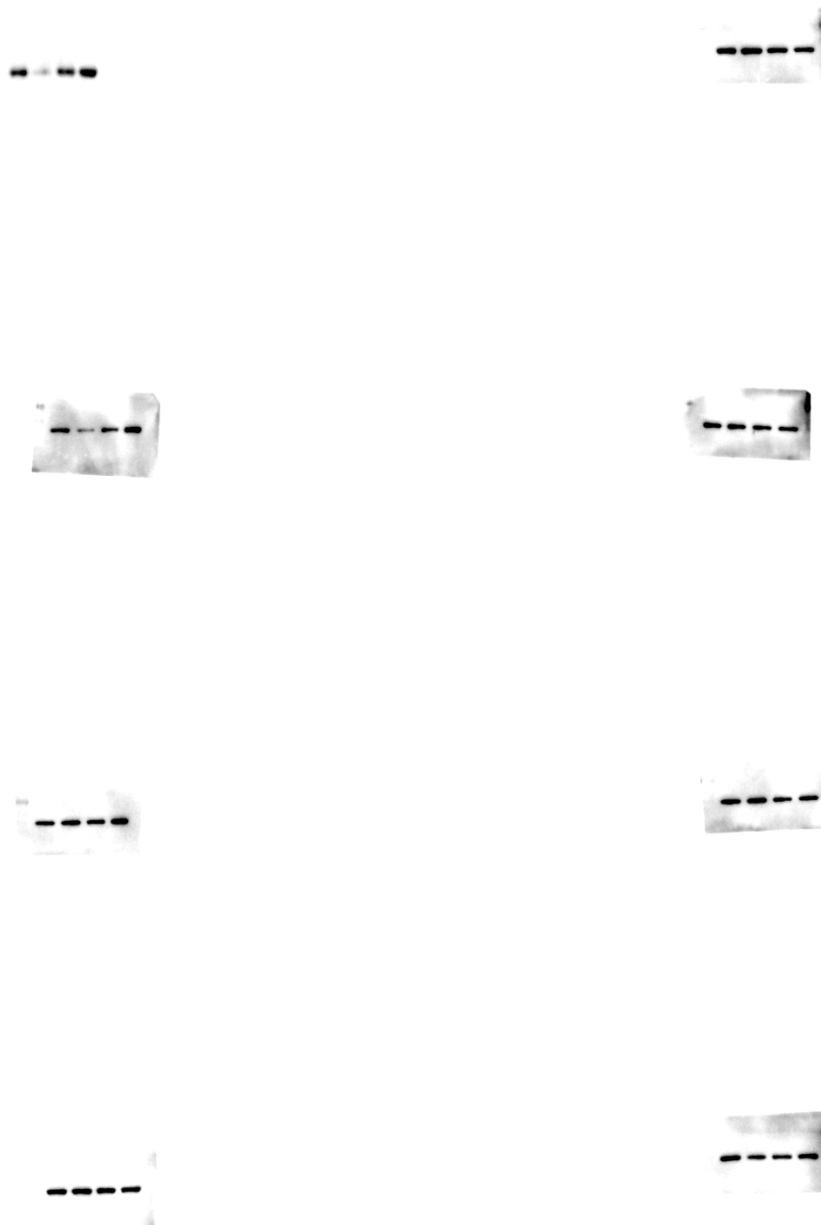

— — — —

— — — —

— — — —

— — — —

— — — —

— — — —

— — — —

— — — —

1

1

1

1

1

1

1

1

1

1

1

---

Fig4

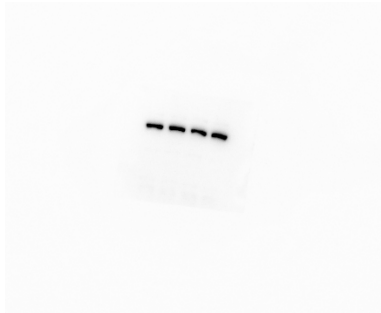

---

---

---

---

---

---

---

--

--

----

----

----

----

----

----

----

----

— — — —

— — — —

— — — —

— — — —

— — — —

— — — —

— — — —

— — — —

— — — —

— — — —

Fig5

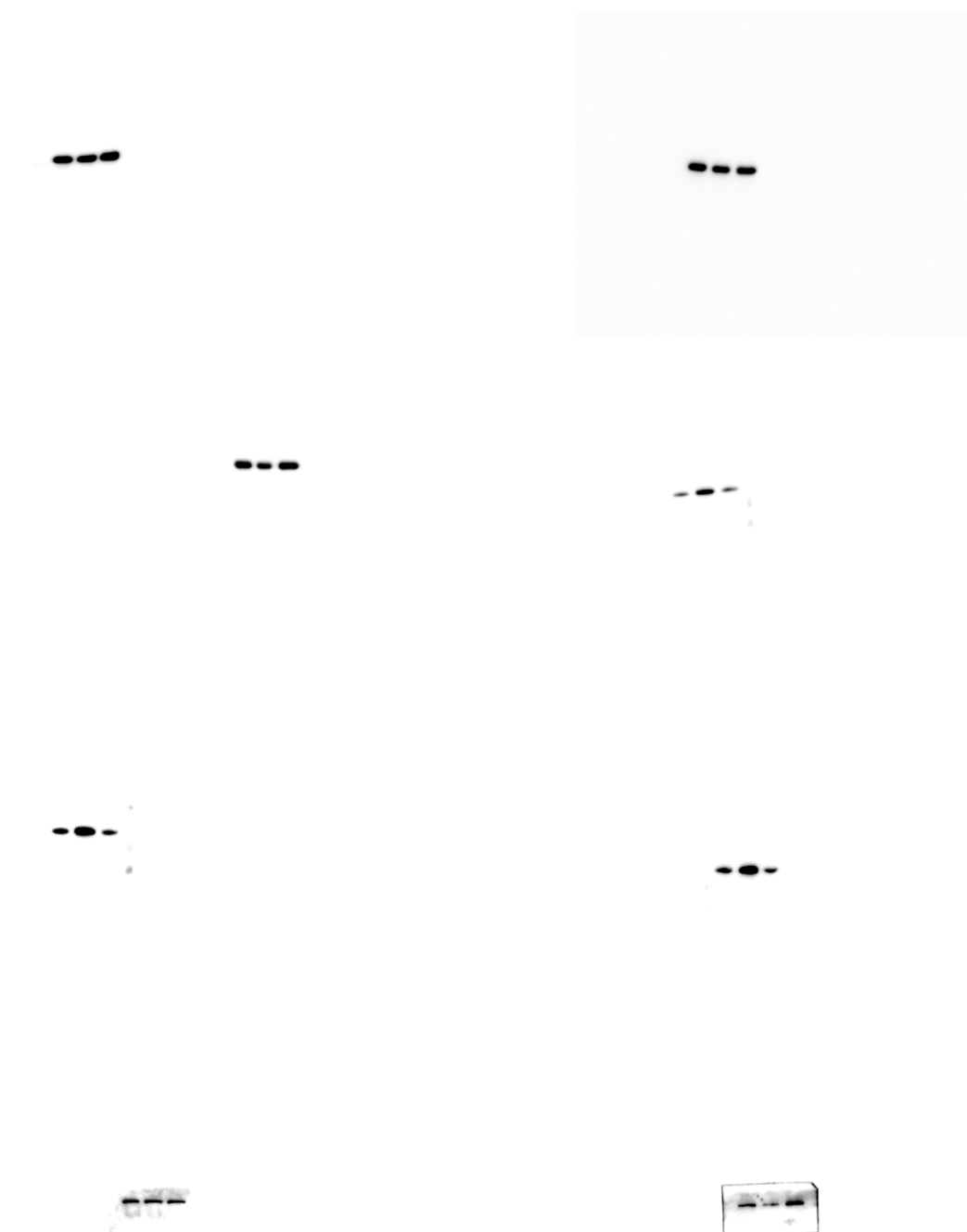

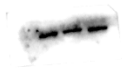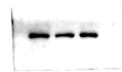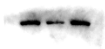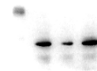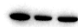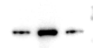

Supplement: Supplementary file 2 — Original data file [file 41420_2024_2163_MOESM2_ESM.pdf]
